# Integrated analysis of metabolites and enzyme activities reveals the plasticity of central carbon metabolism in grape (*Vitis vinifera* cv. Cabernet Sauvignon) berries under carbon limitation

**DOI:** 10.1093/hr/uhae363

**Published:** 2024-12-28

**Authors:** Qian Tong, Yongjian Wang, Regina Feil, John E Lunn, Xiaobo Xu, Yi Wang, Ghislaine Hilbert-Masson, Junhua Kong, Jinliang Chen, Serge Delrot, Bertrand Beauvoit, Zhenchang Liang, Eric Gomès, Yves Gibon, Zhanwu Dai

**Affiliations:** State Key Laboratory of Plant Diversity and Specialty Crops, Beijing Key Laboratory of Grape Science and Enology, Institute of Botany, Chinese Academy of Sciences, Beijing 100093, China; China National Botanical Garden, Beijing 100093, China; University of Chinese Academy of Sciences, Beijing 100049, China; Department of Public Health and Preventive Medicine, Changzhi Medical College, Changzhi 046000, China; State Key Laboratory of Plant Diversity and Specialty Crops, Beijing Key Laboratory of Grape Science and Enology, Institute of Botany, Chinese Academy of Sciences, Beijing 100093, China; China National Botanical Garden, Beijing 100093, China; University of Chinese Academy of Sciences, Beijing 100049, China; Max Planck Institute of Molecular Plant Physiology, Wissenschaftspark Golm, Am Mühlenberg 1, 14476 Potsdam-Golm, Germany; Max Planck Institute of Molecular Plant Physiology, Wissenschaftspark Golm, Am Mühlenberg 1, 14476 Potsdam-Golm, Germany; State Key Laboratory of Plant Diversity and Specialty Crops, Beijing Key Laboratory of Grape Science and Enology, Institute of Botany, Chinese Academy of Sciences, Beijing 100093, China; China National Botanical Garden, Beijing 100093, China; University of Chinese Academy of Sciences, Beijing 100049, China; State Key Laboratory of Plant Diversity and Specialty Crops, Beijing Key Laboratory of Grape Science and Enology, Institute of Botany, Chinese Academy of Sciences, Beijing 100093, China; China National Botanical Garden, Beijing 100093, China; University of Chinese Academy of Sciences, Beijing 100049, China; EGFV, Bordeaux-Sciences Agro, INRAE, ISVV, University of Bordeaux, F-33140 Villenave d'Ornon, France; State Key Laboratory of Plant Diversity and Specialty Crops, Beijing Key Laboratory of Grape Science and Enology, Institute of Botany, Chinese Academy of Sciences, Beijing 100093, China; China National Botanical Garden, Beijing 100093, China; University of Chinese Academy of Sciences, Beijing 100049, China; Center for Agricultural Water Research in China, China Agricultural University, Beijing 100083, China; EGFV, Bordeaux-Sciences Agro, INRAE, ISVV, University of Bordeaux, F-33140 Villenave d'Ornon, France; INRAE, Biologie du Fruit et Pathologie, University of Bordeaux, UMR 1332, F-33140 Villenave d'Ornon, France; State Key Laboratory of Plant Diversity and Specialty Crops, Beijing Key Laboratory of Grape Science and Enology, Institute of Botany, Chinese Academy of Sciences, Beijing 100093, China; China National Botanical Garden, Beijing 100093, China; University of Chinese Academy of Sciences, Beijing 100049, China; EGFV, Bordeaux-Sciences Agro, INRAE, ISVV, University of Bordeaux, F-33140 Villenave d'Ornon, France; INRAE, Biologie du Fruit et Pathologie, University of Bordeaux, UMR 1332, F-33140 Villenave d'Ornon, France; State Key Laboratory of Plant Diversity and Specialty Crops, Beijing Key Laboratory of Grape Science and Enology, Institute of Botany, Chinese Academy of Sciences, Beijing 100093, China; China National Botanical Garden, Beijing 100093, China; University of Chinese Academy of Sciences, Beijing 100049, China

## Abstract

High temperatures increase the sugar concentration of grape (*Vitis vinifera* L.) berries, which can negatively affect the composition and quality of wine, and global climate change is expected to exacerbate this problem. Modifying the source-to-sink ratio of grapevines by selective pruning is a potential strategy to mitigate this. To investigate the effects of low source-to-sink ratio (retaining three leaves per cluster) on carbon metabolism of grape (cv. Cabernet Sauvignon) berries, we conducted an analysis of 42 metabolites and 21 enzyme activities at nine berry developmental stages，as well as transcriptomes from berries grown under two leaves per cluster. The results revealed that the metabolic pathways were coordinately regulated to maintain homeostasis under low source-to-sink ratio conditions. Because of a delay between metabolites and enzyme activities, the metabolites were loosely correlated with enzyme activities, and a lower density of connectivity between them appeared in low source-to-sink conditions. Otherwise, transcripts of the carbohydrate and amino acid metabolism pathways were enriched by carbon limitation. In summary, this integrated analysis reveals a coordinated regulation of various metabolic pathways that maintains the balance of carbon metabolism and ensures survival in challenging environments, highlighting the high metabolic plasticity of grape berries.

## Introduction

Central carbohydrate metabolism during grape berry development is a major determinant of yield and of the levels of primary and secondary metabolites in the fruit at harvest, which influence the alcohol content and organoleptic properties of the wine. This includes sucrose metabolism, glycolysis, TCA cycle (tricarboxylic acid cycle), PPP (pentose phosphate pathway), and amino acid synthesis and catabolism, providing key components that affect fruit flavor, such as sugars that determine sweetness and TCA intermediates that determine acidity. Changes in central carbon metabolite levels are often coordinated during fruit growth and development, ultimately affecting the metabolite composition of fruits [1, 2]. In addition, some metabolites (e.g. malate, sucrose, and trehalose 6-phosphate) can act as signaling molecules regulating metabolic fluxes and gene expression [3–5].

Central carbon metabolism in fruits is influenced by various factors, including environment [6], fruit development stage [7], plant growth regulators [8], and source-to-sink ratios [9, 10]. Modulations of source-to-sink ratio by leaf removal [11, 12], girdling [13], or fruit thinning [14] are common field manipulations that affect carbon metabolism of fruits. Source-to-sink ratio perturbation differentially affects the metabolites of different central carbon metabolism pathways. The effect of low source-to-sink ratio on fruit soluble sugars (sucrose and hexoses) is variable, inducing their decrease in tomato [15, 16], kiwifruit [3], orange [17], and grape [18], or no change in peach [14], *Citrus spp* [19], and tomato [20]. These various responses may be mainly attributed to the gradients of source-to-sink ratios between treatments. Low source-to-sink ratio increased most individual amino acids in tomato [20] and grape [21, 22]. Metabolite profiling of fruits is often limited to the major sugars and organic acids that represent the end points of metabolic pathways. Although this is valuable information, measurements of metabolic intermediates (e.g. sugar phosphates) can provide a better understanding of the underlying metabolic networks and fluxes and therefore insight into how the final metabolite profile of the fruit is determined. However, sugar phosphates (e.g. glucose 6-phosphate (G6P), fructose 6-phosphate (F6P), and uridine diphosphate glucose (UDPG)) were rarely studied under low source-to-sink ratio, and the only studies in tomato [20] and kiwifruit [3] showed that carbon limitation increased the levels of sugar phosphates. In contrast, low source-to-sink ratio had only marginal impacts on TCA cycle intermediates in tomato [20]. The mechanisms underlying the different responses of central metabolism pathways to source-to-sink perturbation in fruit species remain elusive.

Enzyme activities play a key role in the regulation of metabolite levels [23]. The low activity of acid invertase (EC 3.2.1.26) promoted sucrose accumulation under carbon limitation in *Citrus unshiu* [19]. Carbon limitation reduced the activities of the corresponding glycolytic enzymes and TCA cycle enzymes, thus limiting energy depletion in lychee [24]. However, Biais et al. [25] showed that enzyme activities of central carbon metabolism were highly conserved under different source-to-sink ratios in tomato. In addition, the levels of central carbon metabolites are also regulated through transcriptional changes that affect the abundance of enzymes and transporter proteins. A number of major structural genes in central carbohydrate metabolism pathway were downregulated under carbon limitation in tomato [15] and kiwifruit [3]. However, the relative regulatory contributions of enzymes and transcripts to central carbon metabolism still need to be compared and quantified simultaneously. Such multilevel comparison becomes increasingly more feasible with the development of high-throughput technologies to analyze the transcriptome, enzymatic activity levels, and metabolome.

Metabolomic and transcriptomic information are both essential to reveal previously unknown features of metabolic networks [1, 26]. In response to cold stress in tomato, the high expression level of genes related to specific synthesis pathways were consistent with high levels of metabolite accumulation (TCA cycle intermediates and individual amino acids) [27]. However, in Arabidopsis (*Arabidopsis thaliana*) and tomato, the activities of enzymes were strongly correlated with each other, whereas they were only weakly connected to metabolite levels [28, 29]. Moreover, the enzyme activities showed less connectivity to transcripts and even less to metabolites during tomato fruit development [30], possibly as a result of post-transcriptional regulation, including post-translational modification of enzymes and protein turnover [31]. Although several studies have reported two types of data (e.g. transcripts and enzyme activities, or enzyme activities and metabolites), few studies have been carried out to integrate analysis during fruit development to provide a comprehensive insight under different source-to-sink ratios.

Central carbon metabolism is essential for the oenological potential of grape by influencing soluble sugars, organic acids, and amino acids [32]. The rising temperatures and more irregular rainfall associated with global climate change are generally leading to higher soluble sugars and lower organic acids in grape berries [33–35], which leads to excessive alcohol content or sweetness and lack of balance when the fruit is used for wine production. To mitigate these challenges, carbon supply modulation is used to control sugar concentration by modulating the leaf area-to-fruit weight ratio in grape in many wine regions [9]. The leaf area-to-fruit weight ratio can affect the metabolite concentration of the fruit at harvest by altering the transport and distribution of photosynthetic products between leaves and fruit. In the present study, we manipulated carbon supply by removing leaves of *Vitis vinifera* cv. Cabernet Sauvignon fruit-bearing cuttings. We conducted an integrated analysis of metabolites and enzyme activities during fruit development to assess the effects of carbon limitation on berry carbon metabolism. This allowed us to reveal a multilevel regulatory network in response to low source-to-sink ratio in grape berries.

## Results

### Central carbon metabolite profiles of berries under source-to-sink modulation

**Figure 1:**
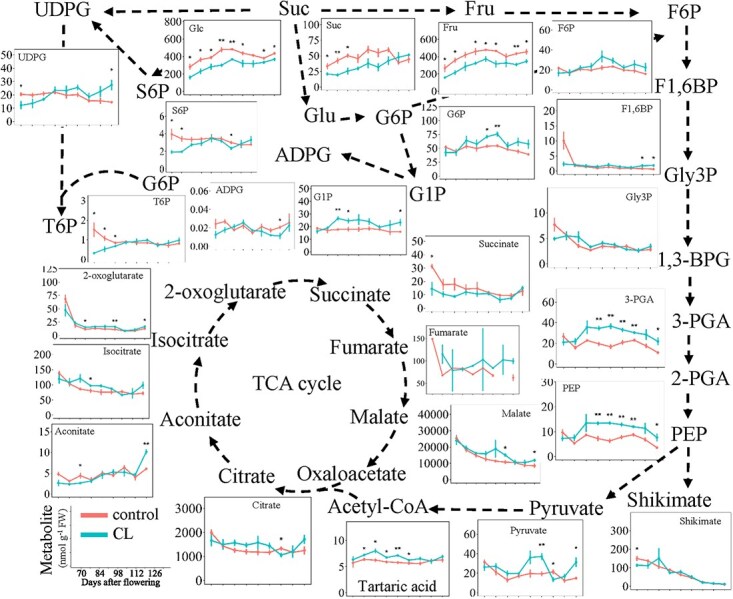
Central carbon metabolite profiles of berries under control and carbon-limited conditions. Metabolites are shown in central carbon metabolic pathways (sucrose metabolism, glycolysis, the TCA cycle) and their developmental profiles are presented alongside (μmol g^−1^ FW for sucrose, glucose, and fructose; and nmol g^−1^ FW for others). CL, carbon limitation. Vertical bars indicate SE (*n =* 3). * indicates *P* < 0.05, and ** indicates *P* < 0.01. Suc, sucrose; Glc, glucose; Fru, fructose; S6P, sucrose 6′-phosphate; UDPG, uridine diphosphate glucose; T6P, trehalose 6-phosphate; G6P, glucose 6-phosphate; G1P, glucose 1-phosphate; ADPG, adenosine diphosphate glucose; F6P, fructose 6-phosphate; F1,6BP, fructose-1,6-bisphosphate; Gly3P, glycerol 3-phosphate; 1,3-BPG, 1,3-bisphosphoglycerate; 3-PGA, 3-phosphoglycerate; 2-PGA, 2-phosphoglycerate; PEP, phosphoenolpyruvate. Metabolites shown in gray were not measured.

Berry weight was not significantly affected under carbon limitation during ripening stages, except during the rapid-growth period (84, 91, and 98 days after flowering (DAF)) when the carbon deficit led to limited berry weight in Experiment 1 (Supplementary Fig. S1a). However, berry weight was decreased during berry ripening stages under carbon limitation in Experiment 2 (Supplementary Fig. S1a). Twenty-four metabolites related to sugar metabolism, glycolysis, and the TCA cycle were analyzed and showed different sensitivities to the source-to-sink modulation in Experiment 1 (Fig. 1 and Supplementary Fig. S2). To consider the potential effects of water budget on metabolite concentrations and quantities, the results were shown both on fresh weight (FW) basis (Fig. 1) and per berry (Supplementary Fig. S2). Since the effect of carbon limitation on berry size was marginal in this experiment, very similar developmental profiles were observed for metabolites when expressed on either basis (Fig. 1 vs Supplementary Fig. S2). Therefore, the results were described hereafter as metabolite concentrations on FW basis. Carbon limitation reduced sucrose concentration by 27%, glucose concentration by 27%, and fructose concentration by 30%. The concentration of most metabolites involved in glycolysis was increased by carbon limitation, including G6P, glucose 1-phosphate (G1P), 3-phosphoglycerate (3-PGA), phosphoenolpyruvate (PEP), and pyruvate. Nucleotide sugars and sugar phosphates, such as UDPG, sucrose 6′-phosphate (S6P) and the sugar-signaling molecule trehalose 6-phosphate (T6P) were transiently decreased by carbon limitation during the first three developmental stages, and then increased or remained unchanged in response to carbon limitation. Adenosine diphosphate glucose (ADPG), F6P, fructose-1,6-bisphosphate (F1,6BP), glycerol 3-phosphate (Gly3P), shikimate, tartaric acid, and all the metabolites involved in the TCA cycle were not clearly influenced by carbon limitation.

**Figure 2:**
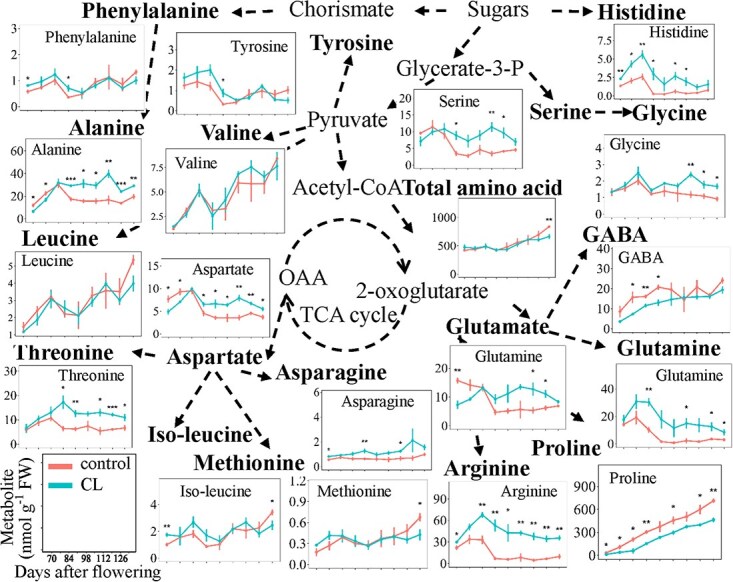
Amino acid concentrations along berry development under control and carbon limited conditions. Vertical bars indicate SE (*n =* 3). * indicates *P* < 0.05, ** indicates *P* < 0.01, and *** indicates *P* < 0.001.

In total, 18 free amino acids were analyzed, and they were differentially affected by carbon limitation during berry development (Fig. 2 and Supplementary Fig. S3). Similarly, quite identical developmental profiles were observed for amino acids, when expressed either on an FW basis or per berry (Fig. 2 and Supplementary Fig. S3). Under carbon limitation, asparagine, arginine, glutamine, and histidine concentrations were higher at all developmental stages. In contrast, proline and γ-aminobutyric acid (GABA) concentrations were significantly decreased under carbon limitation. Alanine, threonine, glycine, and serine were not affected in the first three stages, but were increased under carbon limitation. While glutamate and aspartate were transiently decreased and then increased by carbon limitation, phenylalanine, tyrosine, valine, leucine, iso-leucine, and methionine were hardly influenced by this treatment. Cysteine, lysine, and tryptophan were below the limit of detection. Since proline represents a high percentage (an average percentage of 62.7% in control berries and 40.9% in carbon limited berries) of the total amino acids, its decrease fully counterbalanced the increase in the other amino acids under carbon limitation, resulting in an unchanged concentration of total amino acids between the control and carbon limitation, except for a significant decrease at maturity (Fig. 2 and Supplementary Fig. S3).

Partial least squares discriminant analysis (PLS-DA) was conducted to separate carbon-limited and control berries (Supplementary Fig. S4). The first two discriminatory components explained 45% of sample variation and were able to separate the two populations of berries (Supplementary Fig. S4a). Then the variable importance in projection analysis (VIP analysis, Supplementary Fig. S4B) showed that three amino acids, i.e. glutamine, arginine, and histidine, possessed the largest VIP scores (>2.5) and tended to be higher under carbon limitation. In addition, four amino acids (proline, asparagine, serine, threonine) and one soluble sugar (fructose) also had VIP scores larger than one (Supplementary Fig. S4b). Among them, proline and fructose were lower in berries under carbon limitation, while the reverse was observed for asparagine, serine, and threonine.

### Enzyme activities of berries under source-to-sink modulation

**Figure 3:**
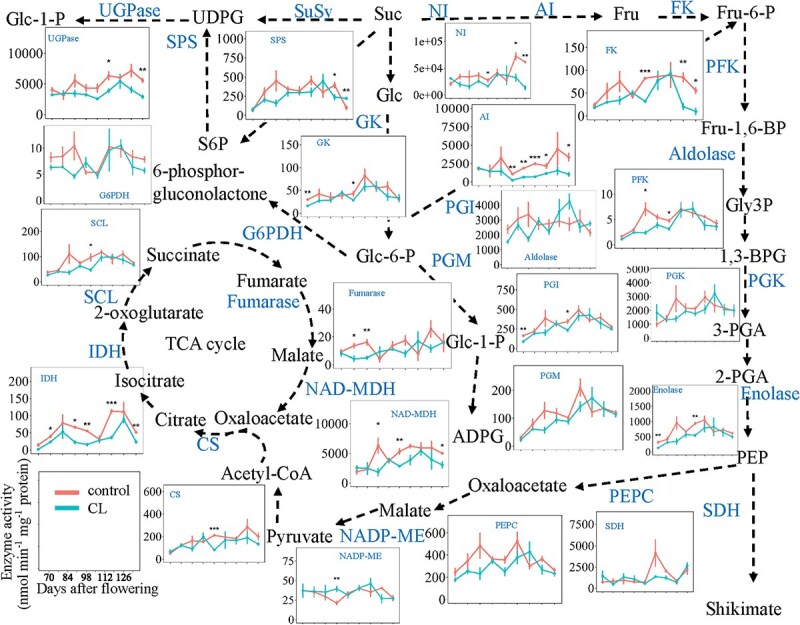
Maximal activities of enzymes in the central carbon metabolic pathways along berry development under control and carbon-limited conditions. The central carbon metabolic pathways are the same as in Fig. 1. Y-axis is in nmol min^−1^ mg^−1^ protein all for enzymes and is in FPKM for mRNA quantities. CL, carbon limited. Vertical bars indicate SE (*n =* 3). * indicates *P* < 0.05, ** indicates *P* < 0.01, and *** indicates *P* < 0.001. SPS, sucrose–phosphate synthase [EC 2.4.1.14]; UGPase, UDP-glucose pyrophosphorylase [EC 2.7.7.9]; NI, neutral invertase [EC 3.2.1.26]; AI, acid invertase [EC 3.2.1.26]; FK, fructokinase [EC 2.7.1.52]; G6PDH, glucose 6-phosphate dehydrogenase [EC 1.1.1.49 EC 1.1.1.363]; GK, glucokinase [EC 2.7.1.1]; PGI, glucose-6-phosphate isomerase [EC 5.3.1.9]; PFK, 6-phosphofructokinase [EC 2.7.1.11]; Aldolase, [EC 4.1.2.13]; PGK, phosphoglycerate kinase [EC 2.7.2.3]; Enolase [EC 4.2.1.11]; SDH, shikimate dehydrogenase [EC 1.1.1.25]; PEPC, phosphoenolpyruvate carboxylase [EC 4.1.1.31]; NADP-ME, malic enzyme [EC 1.1.1.40]; CS, citrate synthase [EC 2.3.3.1]; IDH, isocitrate dehydrogenase [EC 1.1.1.41]; SCL, succinyl-CoA-ligase [EC 6.2.1.5 EC 6.2.1.5]; MDH, malate dehydrogenase [EC 1.1.1.37]; PGM, phosphoglucomutase [EC 5.4.2.2]; fumarase [EC 4.2.1.2].

In total, 21 enzymes involved in sugar metabolism, glycolysis, or the TCA cycle were quantified in grape berries over nine developmental stages under the two source-to-sink ratios. The maximal enzyme activities were expressed both on a protein basis (Fig. 3) and per berry (Supplementary Fig. S5). Since the effects of carbon limitation on berry size were marginal in Experiment 1 (Supplementary Fig. S1), we observed very similar developmental profiles when the enzyme activities were expressed on either basis (Fig. 3 vs Supplementary Fig. S5). Despite the evident differences in metabolite levels of these pathways (Fig. 1), the maximal enzyme activities were less influenced by carbon limitation, showing only slight decreases or no significant differences (Fig. 3). The maximal enzyme activities of acid invertase (AI, EC 3.2.1.26), neutral invertase (NI, EC 3.2.1.26), fructokinase (FK, EC 2.7.1.52), 6-phosphofructokinase (PFK, EC 2.7.1.11), enolase (EC 4.2.1.11), phosphoenolpyruvate carboxylase (PEPC, EC 4.1.1.31), malate dehydrogenase (NAD-MDH, EC 1.1.1.37), isocitrate dehydrogenase (IDH, EC 1.1.1.41), succinyl-CoA-ligase (SCL, EC 6.2.1.4 EC 6.2.1.5), and fumarase (EC 4.2.1.2) were decreased by carbon limitation, particularly during early developmental stages. Moreover, PLS-DA identified that the activities AI and NI had VIP scores larger than one (Supplementary Fig. S4b) and could discriminate berries under carbon limitation. In contrast, the maximal enzyme activities of sucrose-phosphate synthase (SPS, EC 2.4.1.14), glucose 6-phosphate dehydrogenase (G6PDH, EC 1.1.1.49 EC 1.1.1.363), UDP-glucose pyrophosphorylase (UGPase, EC 2.7.7.9), glucokinase (GK, EC 2.7.1.1), glucose-6-phosphate isomerase (PGI, EC5.3.1.9), aldolase (EC 4.1.2.13), phosphoglycerate kinase (PGK, EC 2.7.2.3), shikimate dehydrogenase (SDH, EC 1.1.1.25), phosphoglucomutase (PGM, EC 5.4.2.2), citrate synthase (CS, EC 2.3.3.1), and malic enzyme (NADP-ME, EC 1.1.1.40) were not significantly influenced.

### Transcriptomic responses of grape berries to source-to-sink modulation

We conducted RNA sequencing for 18 cDNA libraries to investigate the transcriptome responses of grape berries to carbon limitation in Experiment 2. For each sample, an average of 6 Gb clean data was obtained, representing an average depth of 140.7x (Supplementary Table S1). An average of 22.75 million clean reads was obtained from 18 samples and mapped to the grape reference genome (PN40024 12X.0 v2.1), with mapping coverage of 87.3%–89.8%. A principal component analysis (PCA) plot revealed a clear separation among samples at three stages as well as a separation between samples of two treatments except two samples under carbon limitation at 79 and 89 DAF (Supplementary Fig. S6), indicating that the results were generally reliable for further differential expression gene (DEG) analysis.

**Figure 4:**
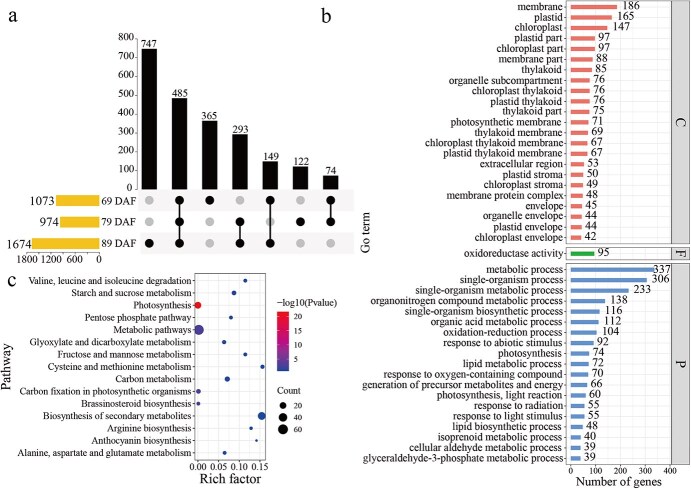
Number and functional enrichment of significantly upregulated genes in response to carbon limitation in grape berries at different development stages. The overlap of significantly upregulated genes in different development stages was visualized with UpSetR (a). UpSetR shows the DEGs overlapped across different berry development stages into intersects. Vertical bars represent the number of genes within each intersect, while the filled circles below show the stages for establishing the intersect. Horizontal bars represent the total number of DEGs for each stage between the two source-to-sink ratios. Functional enrichment analysis for DEGs were conducted with the GO annotation (b) and KEGG pathway annotation (c). In the GO enrichment analysis (b), the C represents cellular component, F for molecular function, and P for biological process.

Carbon limitation led to 3721 upregulated DEGs occurring at one of the three studied developmental stages, with 485 DEGs being commonly upregulated in all the three studied stages (Fig. 4a). GO (Gene Ontology) enrichment analysis for the 485 common DEGs identified 23 significantly enriched GO terms in the category cellular components, 1 in molecular functions, and 26 in biological processes (FDR < 0.01, number > 35) (Fig. 4b). Of note, organic acid, serine family amino acid, aldehyde, and glyceradehyde-3-phosphate metabolic process were enriched by carbon limitation in the upregulated DEGs (Fig. 4b). Furthermore, Kyoto Encyclopedia of Genes and Genomes (KEGG) pathway enrichment analysis also showed that carbohydrate and amino acid metabolism pathways were enriched by carbon limitation among the upregulated DEGs (Fig. 4c).

**Figure 5:**
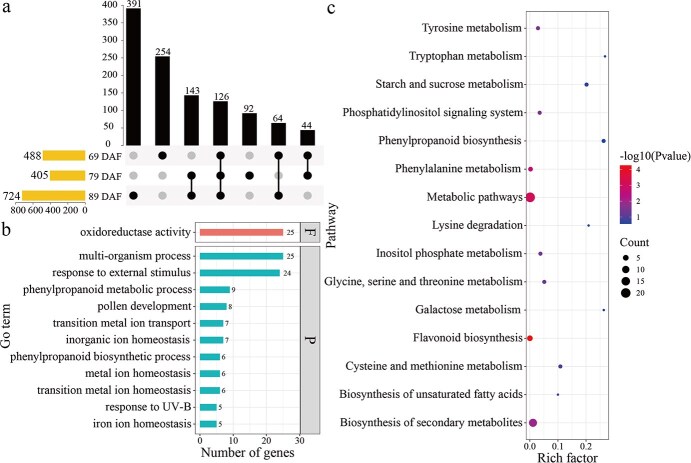
Number and functional enrichment of significantly downregulated genes in response to carbon limitation in grape berries at different development stages. The overlap of significantly downregulated genes at various developmental stages were visualized with UpSetR (a). Functional enrichment analysis for DEGs were conducted with the GO annotation (b) and KEGG pathway annotation (c).

Similarly, carbon limitation led to 1617 downregulated DEGs in at least one developmental stage, with 126 being commonly downregulated for the three developmental stages (Fig. 5a). GO enrichment analysis for the 126 common DEGs identified 11 significantly enriched GO terms in the biological processes category and one in molecular function (FDR < 0.05, number > 0). Phenylpropanoid metabolic and biosynthesis processes were enriched by carbon limitation among the downregulated DEGs (Fig. 5b). Moreover, KEGG pathway enrichment showed that proteins participating in amino acid metabolism, carbohydrate metabolism, and phenylpropanoid and flavonoid biosynthesis were enriched by carbon limitation among the downregulated DEGs (Fig. 5c).

**Figure 6:**
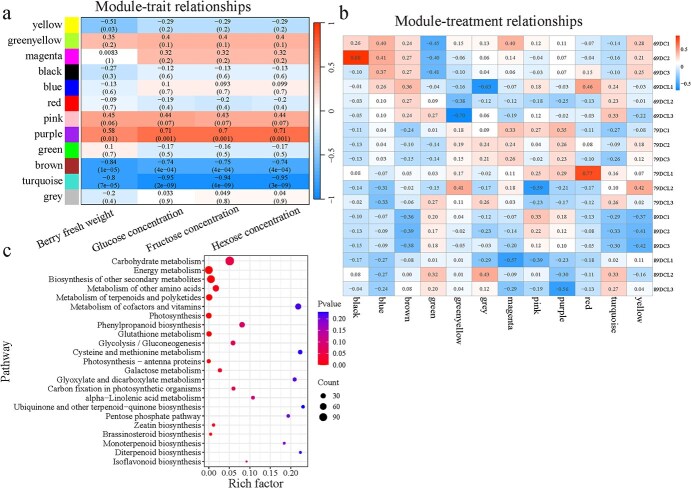
Weighted gene coexpression network analysis of DEGs and five berry traits (berry FW, glucose concentration, fructose concentration, and hexose concentration) of fruit-cutting berries in Experiment 2, and KEGG enrichment of DEGs in ‘turquoise’ module. (a) Module–trait relationship and *P*-values (in parentheses). Color in right panel shows correlation from −1 to 1. In left panel, 12 modules are represented by different colors. (b) Module–treatment relationship and correlation coefficient. The left panel indicates different samples. 69 DC1, 69D, 69 days after treatment; C, control; 1, first repetition. 69DCL1, 69D, 69 days after treatment; CL, carbon limitation; 1, first repetition. (c) Functional enrichment analysis with KEGG pathway annotation in ‘turquoise’ module.

A weighted gene coexpression network analysis was conducted with DEGs and key berry traits of fruit-cutting berries in Experiment 2 (Fig. 6). In total, 12 modules were identified from the analysis of the module–trait relationships (Fig. 6a). Of these modules, the ‘turquoise’ module was highly correlated with hexose concentration (*r* = −0.95, *P* = 3 × 10^−9^). Compared to the other modules, this ‘turquoise’ module showed the highest correlation with all the other four traits. Meanwhile, the ‘turquoise’ module was also specifically associated with treatments (negative correlation existed with control and positive correlation existed with carbon limitation) (Fig. 6b). In total, 3150 genes were identified in the ‘turquoise’ module, and most of these genes were enriched in carbohydrate metabolism pathways and amino acid metabolism pathways with KEGG enrichment analysis (Fig. 6c).

### Correlations between metabolites and enzyme activities in grape berries under source-to-sink modulation

**Figure 7:**
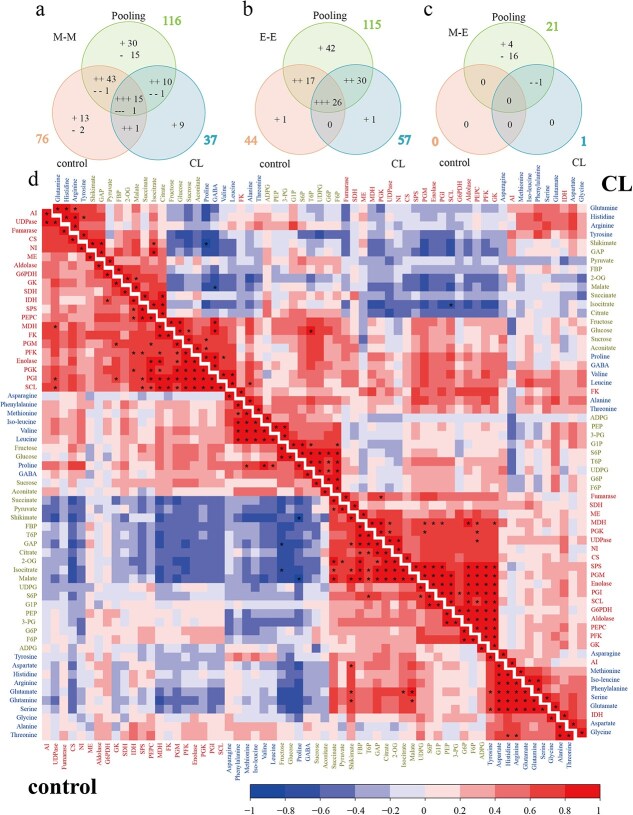
Number and correlation matrix of metabolites and enzyme activities at nine development stages from berries under control (left bottom) or carbon-limited condition (CL, top right, *n =* 3). Correlation analysis was also conducted by pooling data from both CL and control berries, and the number of significant correlation pairs (*P* < 0.00001) between central carbon metabolites (M, central carbon metabolites and amino acids) and enzymes (E) is shown in the Venn diagram, with ‘+’ for positive and ‘−’ for negative correlations, respectively. (a–c) The Venn diagrams show the number of correlations identified from data of control, CL, and the pooled data. M-M: the correlation between metabolite and metabolite, M-E: the correlation between metabolite and enzyme activity. (d) The magnitude and direction of the Pearson correlation coefficient are represented by different colors as indicated in the color key. Central carbon metabolites, free amino acids, and enzyme activities were highlighted with different colors and grouped within the correlation matrix with k-mean clustering.

To investigate the relationship among 42 metabolites and 21 enzyme activities, correlation analyses were conducted by pooling data from both carbon-limited and control berries, or by analyzing the data from carbon-limited or control berries separately, across the nine developmental stages (Fig. 7). There were 120 significant correlations (*P* < 0.00001) in control berries, including 76 correlations in metabolite–metabolite pairs (Fig. 7a) and 44 correlations in enzyme–enzyme pairs (Fig. 7b). Among these, most pairs showed positive correlations and the only four negative correlations were between metabolites, including proline/shikimate (*r* = −0.95), proline/malate (*r* = −0.83), fructose/GAP (*r* = −0.77), and fructose/iso-citrate (*r* = −0.77) (Fig. 7a and d).

There were 95 significant correlations (*P* < 0.00001) under carbon-limiting conditions, including 37 in metabolite–metabolite pairs (Fig. 7a), 57 in enzyme–enzyme pairs (Fig. 7b), and only 1 between metabolite and enzyme (Fig. 7c). All of the significant positive correlations were found in the metabolite–metabolite or enzyme–enzyme pairs, including 35 positive correlations in metabolite–metabolite pairs (Fig. 7a) and 56 positive correlations in enzyme–enzyme pairs (Fig. 7b). In the metabolite–metabolite pairs, two were negative, i.e. proline/shikimate (*r* = −0.80) and malate/GABA (*r* = −0.75) (Fig. 7a and d). Only one significant correlation was observed between metabolites and enzymes, between isocitrate and SCL (*r* = −0.77) (Fig. 7c and d). The number of significant correlations under control carbon supply was more than those under carbon limitation. The number of significant correlations between metabolites in control berries (76 correlations in control berries) was higher than in carbon-limited berries (37 correlations in carbon-limited berries, Fig. 7a), while the number of significant correlations between enzyme activities in the controls (44 correlations in control berries) was less than in carbon-limited samples (57 correlations in carbon-limited berries, Fig. 7b).

When pooling the data from both carbon supply levels, the significant correlations were increased to 251 (*P* < 0.00001), including 116 correlations in metabolite–metabolite pairs (Fig. 7a, Supplementary Fig. S7), 115 positive correlations in enzyme–enzyme pairs (Fig. 7b), and 21 correlations between metabolites and enzymes (Fig. 7c). There were 15 significant positive correlations and one negative correlation between metabolites and metabolites that were seen in both the individual datasets (control and carbon limitation) as well as in the pooled data (Fig. 7a and d, Supplementary Fig. S7, Supplementary Table S2). There were 26 correlations in common for enzyme–enzyme pairs among the control, carbon-limitation, and the pooled data (Fig. 7b and d, Supplementary Fig. S7, Supplementary Table S2).

## Discussion

### Rebalancing of metabolic pathways to maintain internal homeostasis under carbon limitation

Plants with fleshy fruits can provide nutrients to frugivores as rewards for promoting seed spreading [36, 37] and therefore have evolved capability to adapt their central carbohydrate metabolisms to various stresses and environmental perturbations [38–40]. These external perturbations often modify the source-to-sink ratio and cause carbon limitation. Moreover, central carbohydrate metabolism is very dynamic over the course of grape berry development, with major adjustments of the metabolic network to meet the changing demands for growth and sugar accumulation [2]. Consistent with previous findings [18, 21, 41–45], we found that the hexose concentration was decreased by carbon limitation during the ripening period (Fig. 1). Sugar phosphates (S6P and UDPG) were strongly decreased during the early stage before recovering to the levels seen in carbon-sufficient controls in the middle and late stages of ripening period. Under carbon-limiting conditions, several glycolytic intermediates (G6P, 3-PGA, PEP, and pyruvate) were increased in middle and late ripening stages. The carbon limitation-induced increase in 3-PGA was likely caused by a reduced catabolism associated with decreased enolase activity, while its synthesis is not affected as indicated by the unaltered PGK activity. Similarly, the increase of PEP concentration under carbon-limited conditions might mainly result from reduced consumption of PEP for anaplerotic flux of carbon into the TCA cycle, which is suggested by the lower PEPC activities. Although the maximal activities of a few enzymes (fumarase and MDH) of the TCA cycle were decreased by limited carbon supply, the TCA cycle intermediates were hardly affected by this treatment. This suggests that changing the maximal capacity of these enzymes may not affect the metabolic flux.

Concomitantly, carbon limitation increased some amino acids, especially the glycolytic pathway-derived amino acids (serine, glycine, and alanine) and the TCA cycle-derived amino acids (glutamate, glutamine, arginine, aspartate, threonine, and asparagine) (Fig. 3). These increases in amino acid content might be attributed to a general decrease in protein synthesis in carbon-limited berries. Previous studies have shown that protein synthesis has a high energetic cost and that translation is sensitive to carbon availability, which is sensed and regulated via the SnRK1 and TOR pathway [46]. Under carbon-limited conditions, the decreased carbon availability will be sensed and lead to restriction of translation, resulting in accumulation of amino acids that are not being used for protein synthesis [47, 48]. Alternatively, the high levels of amino acids may also compensate the carbon deficit to maintain homeostasis in berries, since amino acids can act as compatible osmolytes to maintain osmotic homeostasis under stress conditions [49]. In fact, plants keep constantly adapting to maintain cellular homeostasis by controlling the flux of metabolites to ensure their dynamic equilibrium [50]. Apart from this, the development and ripening of fruits will be delayed because of the lack of carbon supply [51]. Previous results showed that the accumulation of anthocyanins was decreased in berries under low source-to-sink ratio [18, 21, 22]. Therefore, a negative correlation exists between amino acid and anthocyanin concentrations [52, 53]. It strengthens the idea that the inhibition of fruit growth and development due to carbon limitation blocks the synthesis of specialized metabolites and ultimately leads to the accumulation of amino acids. Proline, the most abundant amino acid in grape pulp [53], was decreased under low source-to-sink ratio. In addition, proline, an important osmolyte, generally accumulates in response to osmotic stress [54, 55], and the higher level of proline may reflect the response to osmotic stress induced by higher sugars (almost 1 M, Fig. 3) in the berries under high carbon supply condition. On the other hand, the lower sugar levels under carbon limitation may cause lower or no osmotic stress and consequently, lower proline amounts were accumulated.

Under low source-to-sink ratio, the T6P concentration was decreased only in the early stage in grape berries (Fig. 1), as reported in kiwifruit [3]. T6P is a vital sugar signal influencing plant metabolism and development [56–58], and its concentration is often tightly correlated with that of sucrose [59, 60]. However, we still did not detect strong correlations between T6P and sucrose in grape berries under two carbon supply levels, as previously reported [2]. Subcellular compartmentation is one explanation for the apparent lack of correlation between T6P and sucrose. T6P is synthesized in the cytosol and is thought to respond to changes in the nucleo-cytosolic pool of sugars [56–58], so changes in sugar concentration in the very large vacuoles of grape berry cells can mask the changes in the smaller nucleo-cytosolic pool. Moreover, the settings of the sucrose–T6P nexus might be altered during the course of grape berry development, to match the different metabolic status and needs of the cells during cell division, expansion, sugar accumulation, and ripening phases.

### Central carbohydrate metabolites are loosely correlated with enzyme activities

Although some metabolites were affected by carbon limitation, the related maximal enzyme activities were essentially unchanged (Fig. 3), which was consistent with a previous study of the effect of shading on tomatoes [25]. This suggests that carbon limitation alters the accumulation of some metabolites most likely through changing the amounts of proteins and/or their post-translational modifications [61, 62]. Moreover, the enzyme activities measured represent the maximal capacity of that enzyme with saturating substrate concentrations [62]. In central carbohydrate metabolism, the actual enzyme activities *in vivo* are likely to be <50% of the maximal activities due to subsaturating substrate concentrations and the presence of inhibitors and post-translational modifications *in vivo* [63]. Thus, it is possible to find altered actual metabolic fluxes in the absence of significant changes in maximal enzyme capacities. In addition, the post-translational modifications and transport or compartmentation of metabolite may also affect the correlations between metabolites and enzyme activities. In future, proteomics and fluxomics approaches are therefore required to further clarify the biochemical basis of the observed changes in metabolite levels.

Central carbohydrate metabolites strongly correlated with each other, and the measured enzyme activities also frequently correlated with each other. It is noteworthy that the correlations among metabolites were much lower under low source-to-sink ratio condition than under control condition, and a large proportion of the correlations showed positive links. This indicates that carbon limitation may alter the poise among metabolite levels and alter the coordination between them, in agreement with previous work on tomato [20, 64, 65]. On the other hand, metabolite abundance and enzyme activities were less correlated (Fig. 7), which also agrees with a previous work in tomato [29]. Moreover, the connectivity between metabolites and enzyme activities depended on the source-to-sink ratio as previously reported [20, 66].

### Limitation of the current study

Despite the insights described above in this study, it is worth noting that there are still some limitations requiring further exploration. Firstly, we used biological samples by pooling various berries to form one replicate. Although this strategy has been extensively applied in fruit research, it may mask the impacts of intrinsic heterogeneity of the berries within a cluster, between clusters or at different levels [67, 68], even though it sticks to the reality of the vineyard situation. Some attempts in the literature have developed single-berry or berry-by-berry sampling strategy for metabolic analysis [69, 70], and may serve a promising alternative sampling strategy, particularly when novel methodologies will allow to conduct multiomics analysis with smaller amounts of sample. Currently, a single berry does not provide enough materials for the analysis of metabolites and enzymes, and transcripts simultaneously. Secondly, our measurements of metabolites and enzyme activities were performed on berries from one experiment, while the transcriptome analysis was conducted on berries from a repetition of the experiment. Great care was taken to ensure that the two experiments were performed in the same way and we used the hexose content of the berries, which was measured in both experiments, to normalize the data to make them as comparable as possible. Moreover, these kinds of leaf-to-fruit ratio experiments have been conducted multiple times, and highly reproducible responsive patterns were observed from different batches of experiments [21, 22, 45].

### Conclusion

In conclusion, the metabolic pathways are coordinately regulated to maintain osmotic balance and homeostasis in berries under carbon-limited conditions. In the low source-to-sink ratio, several glycolytic intermediates were elevated, while the TCA cycle intermediates did not change significantly, as the glycolytic pathway might be activated to provide adequate substrate levels for the TCA cycle to supplement carbon requirement. The increased concentrations of several amino acids might be attributed to the lower rates of protein synthesis under low carbon supply conditions, where amino acids are not being used for protein synthesis and accumulate in large concentrations. Furthermore, amino acids may also serve to compensate for the carbon deficit and maintain osmotic balance. Although some metabolites were changed, the maximal activities of most enzymes were relatively unresponsive to carbon limitation. Moreover, the density of correlation between metabolites and the maximal enzyme activities depended on the source-to-sink ratio. This may reflect the plasticity of metabolic regulation in grape berries. Under non-carbon-limited conditions, metabolism operates with few restrictions on fluxes, thereby maximizing rates of growth and sugar accumulation. In contrast, under carbon-limiting conditions, there will be stronger constraints on growth, requiring metabolism to be regulated more tightly to avoid imbalances between production of energy and building blocks (e.g. amino acids), both of which are needed for growth.

In the future, proteomic studies, including analysis protein modifications, and measurements of metabolic fluxes could provide further insights into the structure and regulation of the metabolic network. Such knowledge could help viticulturalists to modify source-to-sink ratios and breeders to develop varieties that are better adapted to high temperatures, thereby mitigating the potential impact of climate change on grape production.

## Materials and methods

### Plant materials

#### Experiment 1:

The 1-year-old fruiting-cuttings (*V. vinifera* cv. Cabernet Sauvignon) with only one branch and one cluster per plant were cultured as detailed in Mullins and Rajasekaran [71] in a semicontrolled greenhouse. The environmental conditions, plant management, and sampling procedures were detailed in Bobeica et al. [18]. Briefly, 30 plants were randomly distributed into three blocks with 10 plants per block. The source-to-sink ratios were set by partial defoliation at ~1 week before veraison, which was determined as the date of midcoloration of the clusters and corresponded to 63 DAF. Before the treatment, we trimmed each fruit cutting to retain 12 leaves, and then treated with the leaf removal to carbon limitation. In each block, one set of five plants retained 12 leaves per cluster (control) and the other retained three leaves immediately above the cluster per plant (carbon limitation, CL). Grape berries were sampled at nine 7-day intervals from 70 to 126 DAF. Fifteen berries were sampled from three individual plants as a biological replicate. Sampled berries were rapidly frozen in liquid nitrogen. Berries were slightly thawed to separate the skin and pulp, and the seeds were then removed from the pulp. The pulp was ground to a fine powder separately in liquid nitrogen and stored at −80°C until used for analysis of metabolites, enzyme activities, and amino acids.

#### Experiment 2:

The 1-year-old fruiting-cuttings were cultured as in Experiment 1 except that carbon limitation was imposed by retaining only two leaves per cluster. Similar to Experiment 1, 30 plants were randomly distributed into three blocks with 10 plants per block. Both treatments were conducted at ~1 week before veraison, which was determined as the date of midcoloration of the clusters and corresponded to 59 DAF, and berries were sampled at 69, 79, 89, 113, and 128 DAF. At each sampling date, four berries were uniformly sampled from each cluster, and finally 20 berries were taken from five clusters as a biological replicate, with a total of three biological replicates for each treatment. Sampled berries were rapidly frozen in liquid nitrogen and stored in −80°C. After seed removal, the berries were ground to a fine powder in liquid nitrogen. The berries sampled at 69, 79, and 89 DAF were used for transcriptomic analysis by RNA sequencing (RNA-Seq).

### Analysis of central carbon metabolites

Aliquots of 15–20 mg pulp powder from Experiment 1 were extracted with chloroform/methanol [60]. The extracts were used to measure the central carbon metabolism intermediates (e.g. sugar phosphates, glycolysis intermediates, and TCA cycle intermediates). The concentrations of these metabolites were measured using high-performance liquid chromatography (HPLC) with an ICS 3000 chromatograph (Dionex Corporation, Sunnyvale, USA) coupled to a QTrap 6500 triple quadrupole mass spectrometer (AB Sciex, Foster City, USA) as detailed in [60] with modifications as described in Figueroa et al. [72]. The integration of the chromatograms was made with the Analyst software (AB Sciex).

### Analysis of soluble sugars and amino acids

Aliquots of 500 mg pulp powder from Experiments 1 and 2 were sequentially extracted with 2 ml 80% (v/v) and 2 ml 50% (v/v) ethanol, and the ethanolic extracts were pooled, dried *in vacuo*, and dissolved in 2.5 ml deionized water. The extracts were used to determine soluble sugars and amino acids. The concentrations of soluble sugars (sucrose, glucose, and fructose) were measured enzymatically as detailed in Bobeica et al. [18]. The concentrations of amino acids were measured by HPLC (Thermo Fisher Scientific, Waltham, USA) with an FL 3000 dual monochromator detector (Thermo Detector Products, San Jose, USA) after derivatization with an AccQ-Fluor Reagent Kit (Waters, Milford, USA) according to Pereira et al. [73] and Guan et al. [53]. The separation column was a Nova-Pack C18 AccQ-Tag (WAT052885, Waters, Milford, USA) column at 37°C with elution at 1 ml min^−1^.

### Analysis of enzyme activities

Aliquots of 20 mg pulp powder from Experiment 1 were extracted by vigorous vortexing in 500 μl extraction buffer as detailed in Gibon et al. [62] and Biais et al. [25]. The extracts were diluted as needed (Supplementary Table S3) and 2 or 5 μl diluted extract were added to a 96-well microplate at 4°C. After addition of 40 μl reaction mix, and gentle shaking, the plates were incubated at 25°C for 5 min. Five μl starter solution were added, mixed, and set aside for a defined time (the time depending on the assay). At this time, 20 μl stop solution were added, and incubated for a further 10 min at room temperature. Finally, after addition of 20 μl neutralizing solution and 50 μl determination mix, the absorbance value at 340 or 570 nm was immediately monitored with a SAFAS MP96 microplate reader (Monaco) at the HiTMe platform [25] (Supplementary Table S3).

### Transcriptomic analysis by RNA-seq

Total RNA of samples from Experiment 2 was extracted using a plant total RNA isolation kit (Biomarker, Beijing, China). Library construction and transcriptome sequencing were provided by Biomarker Technologies (https://www.biomarker.com.cn) (Beijing, China). The raw count data were preprocessed by removing adapters (TruSeq3-PE.fa:2:30:10) and discarding low-quality sequences (LEADING:3 TRAILING:3 SLIDINGWINDOW:4:15 MINLEN:36) to obtain clean data with Trimmomatic software v.0.39 [74]. The clean sequences were mapped to the grape reference genome PN40024 12X. v2.1 by STAR v.2.7.9a [75]. The expression levels were quantified with FPKM (fragments per kilobase of transcript per million mapped reads) values by RSEM v.1.3.1 (https://github.com/deweylab/RSEM). By using DESeq2 R package v.1.34.0 [76], the DEGs (differentially expression genes) were filtered using the following cutoffs: *P*-value (*P* < 0.05) and fold change (fc > 2). Gene annotation was accomplished with AnnotationHub R package v.3.2.0 (https://bioconductor.org/packages/release/bioc/html/AnnotationHub.html).

The common significant difference genes among different developmental stages were identified with TBtools v.1.098685 [77]. GO enrichment of common genes was analyzed with AgriGo v.2.0 [78]. The KEGG pathways enrichment was used for pathway mapping with KAAS v.2.1 [79].

### Data analysis

All the data analysis and graphing were performed in R software [80].

## Supplementary Material

Web_Material_uhae363

## Data Availability

All data supporting the findings of this study are available within the paper and within its supplementary materials. The RNA-seq data can be found in National Genomics Data Center under accession number CRA007714.
