# Supplementary material for: Integrated analysis of metabolites and enzyme activities reveals the plasticity of central carbon metabolism in grape (Vitis vinifera cv. Cabernet Sauvignon) berries under carbon limitation
Source: Hortic Res. 2024 Dec 28;12(4):uhae363. doi: 10.1093/hr/uhae363 (PMC11891482; doi:10.1093/hr/uhae363)
Supplement: Web_Material_uhae363 [file web_material_uhae363.zip › HR-2024-988-Supplementary Figure.docx]

**Supplementary Figure**


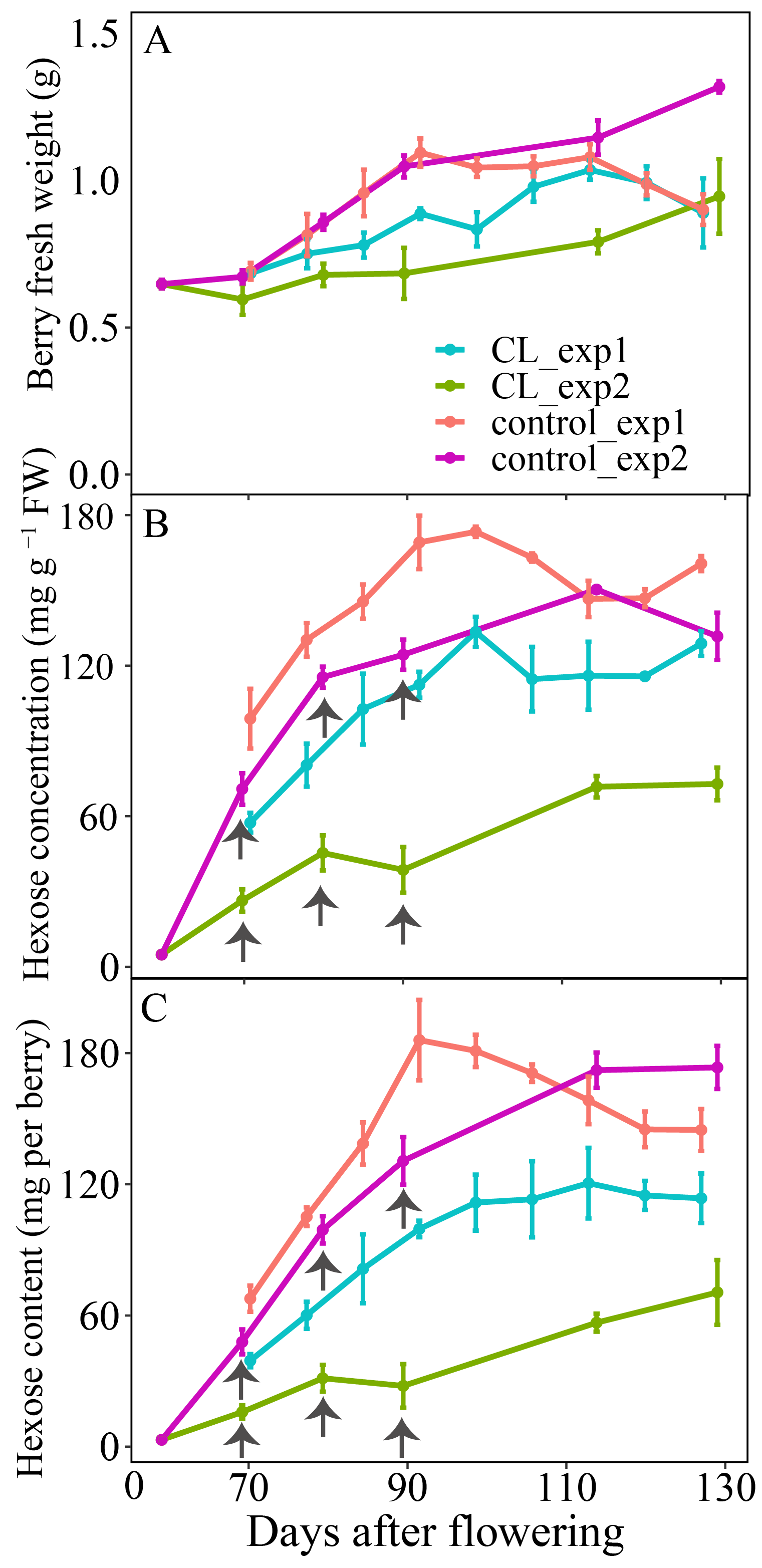


**Supplementary Figure S1**. Berry fresh weight (A), hexose concentration (B) and hexose content (C) under control and carbon limitation during different ripening stages in experiment 1 and experiment 2. CL: carbon limitation. exp1: experiment 1; exp2: experiment 2. The grey arrows indicate the dates of sampling for RNAseq data in the experiment 2. Vertical bars indicate SE (*n*=3).


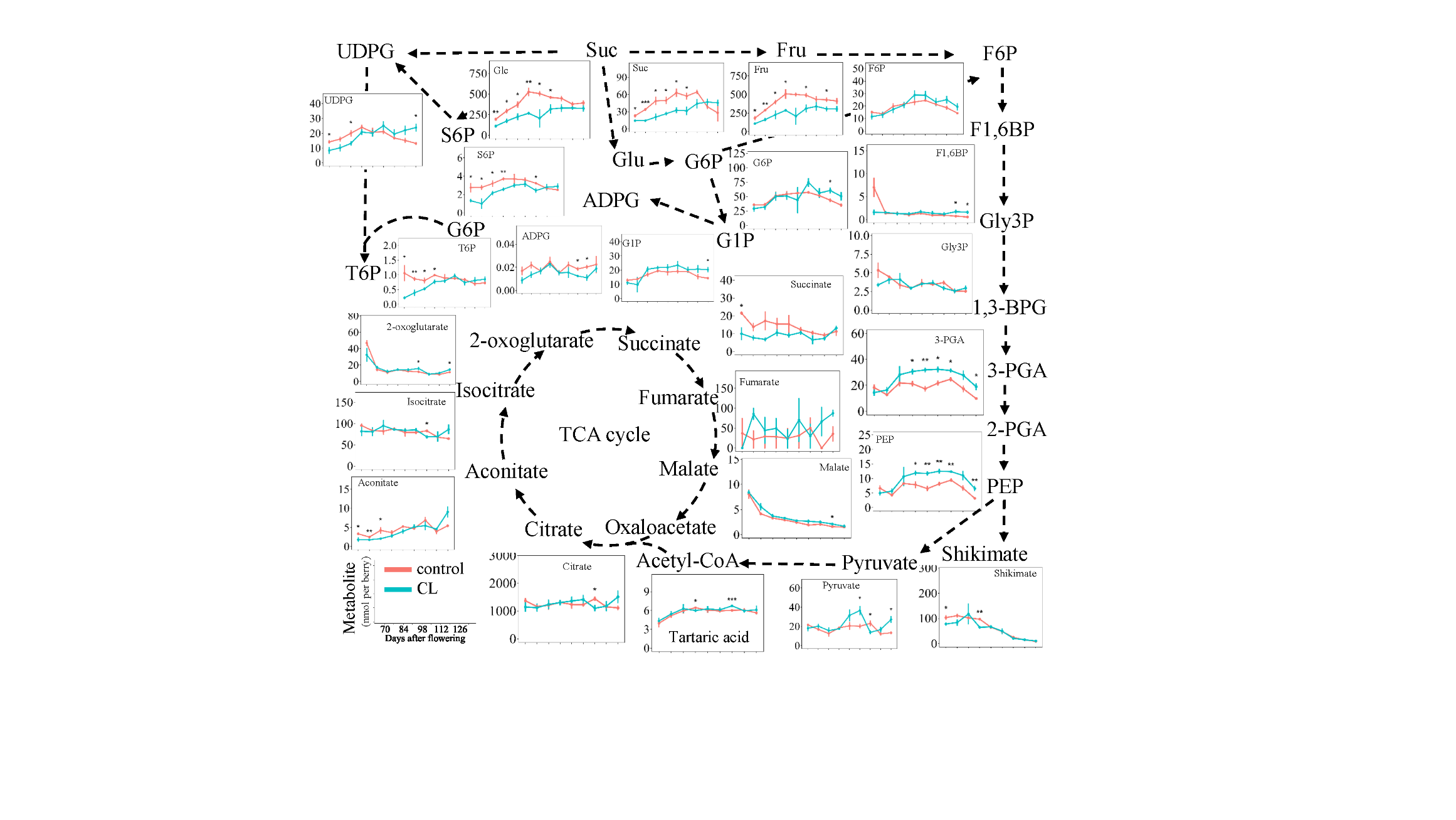


**Supplementary Figure S2.** Central carbon metabolite profiles of berries under control (red line) and carbon limited conditions (blue line). Metabolites are shown in central carbon metabolic pathways (sucrose metabolism, glycolysis, the TCA cycle) and their developmental profiles are presented alongside (μmol per berry for sucrose, glucose and fructose; and nmol per berry for others). CL, carbon limitation. Vertical bars indicate SE (*n=*3). * indicates *P* < 0.05, ** indicates *P* < 0.01, and *** indicates *P* < 0.001. The abbreviations represent the same metabolites as in Fig. 1. Metabolites shown in gray were not measured.


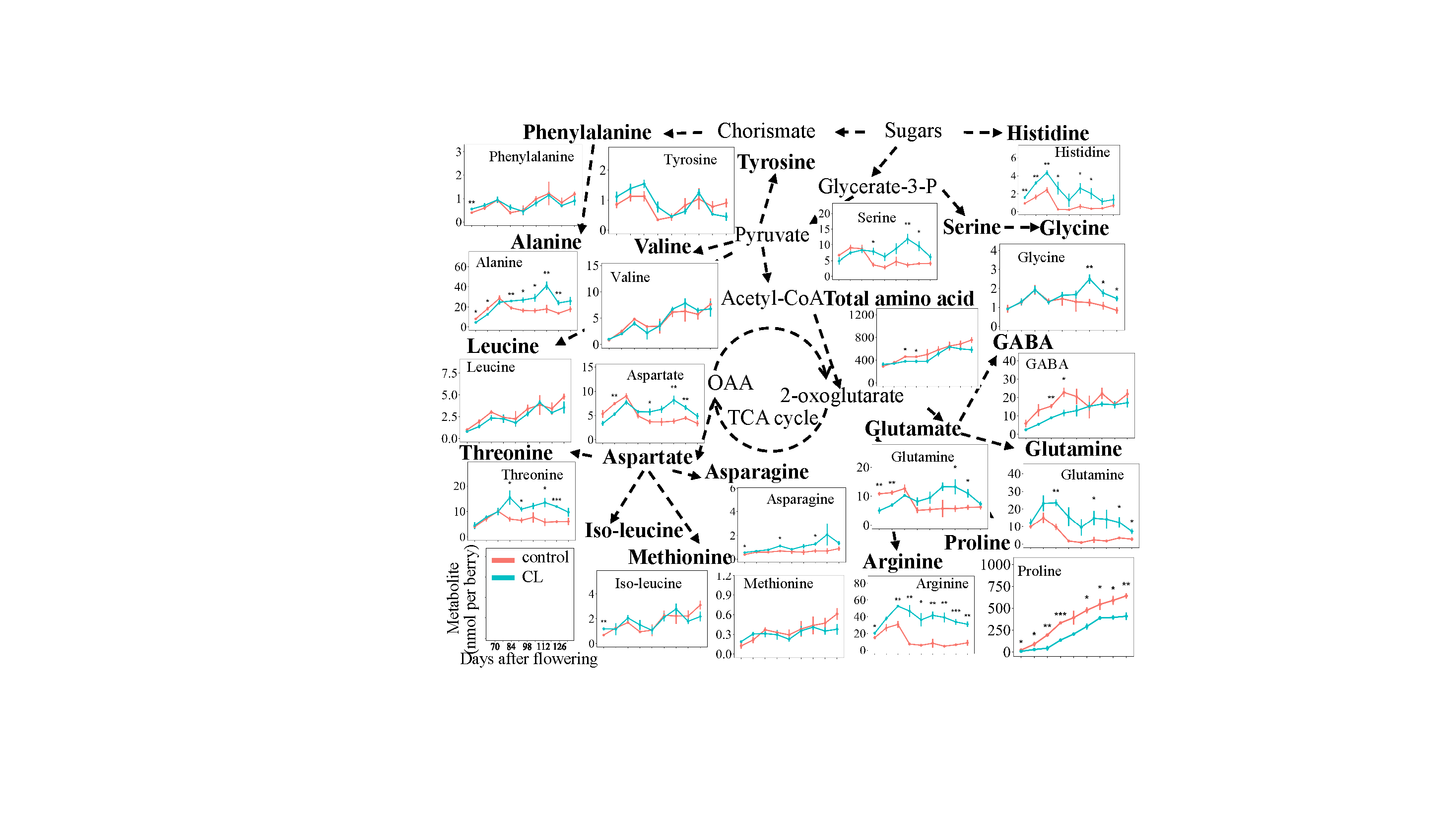


**Supplementary Figure S3. Amino acid concentrations along berry development under control and carbon limited conditions.** CL, carbon limited. Vertical bars indicate SE (*n=*3). * indicates *P* < 0.05, ** indicates *P* < 0.01, and *** indicates *P* < 0.001.


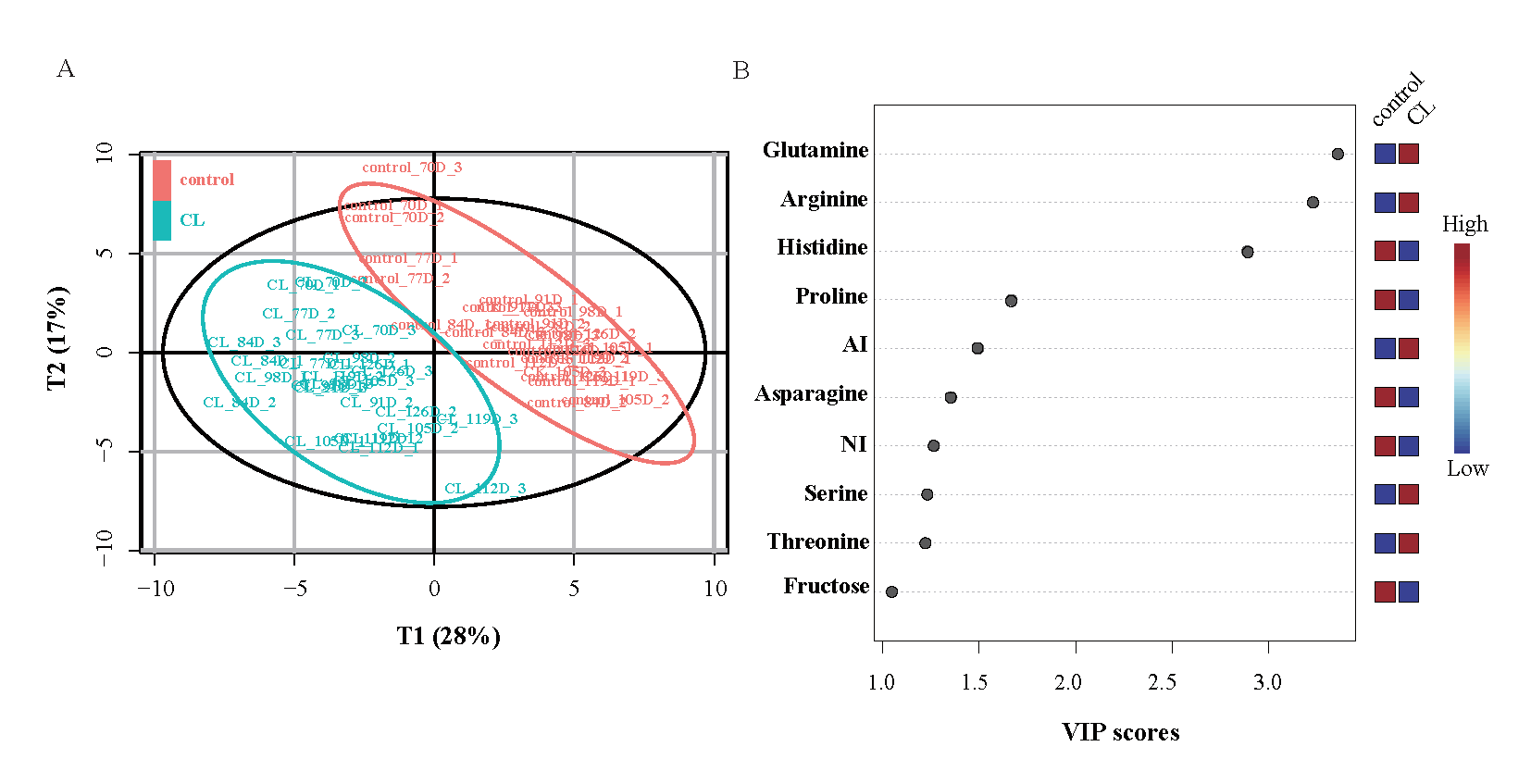


**Supplementary Figure S4**. Discriminant analysis of berries grown under carbon limitation (CL) or control conditions.


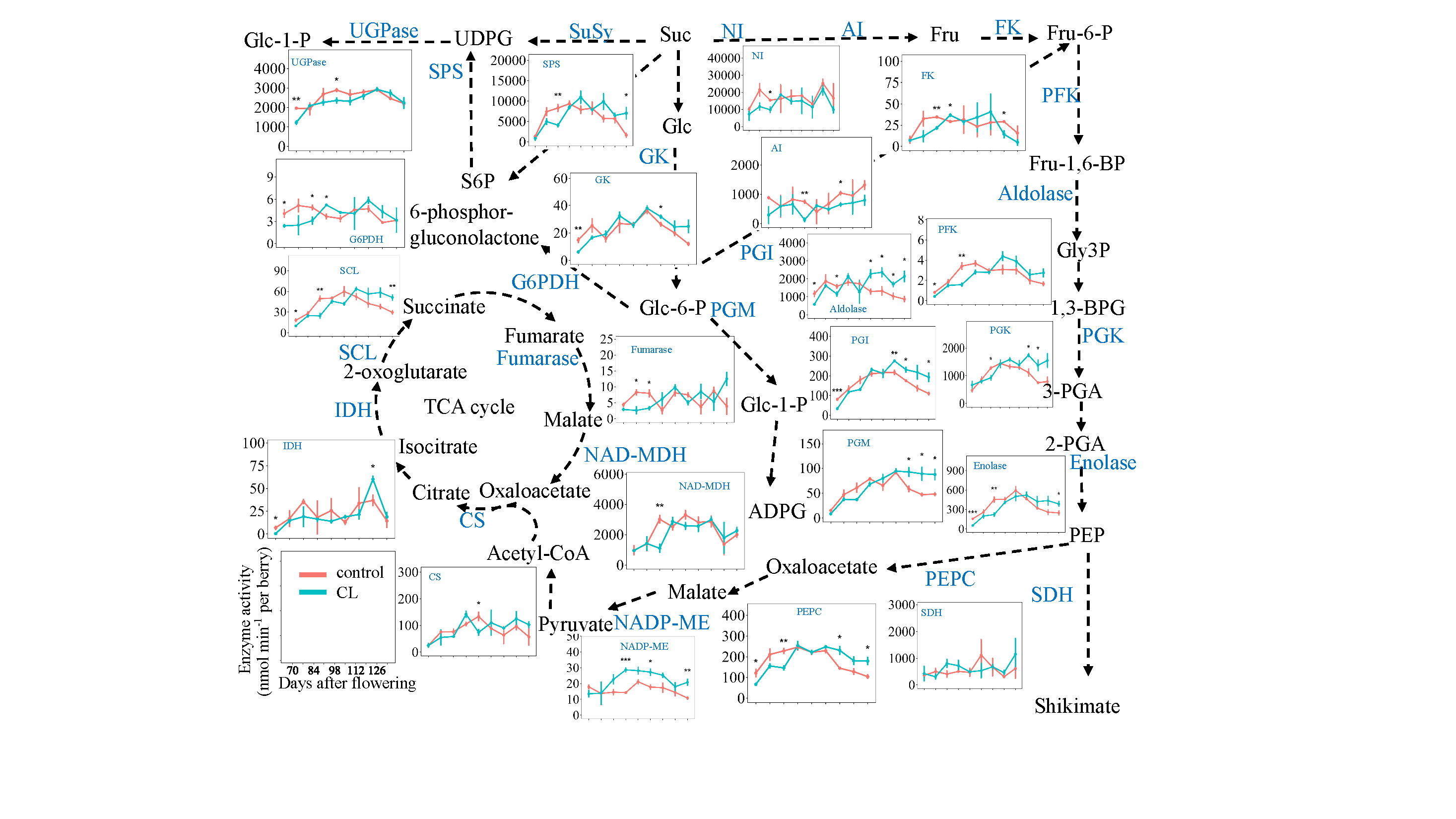


**Supplementary Figure S5**. Maximal activities of enzymes in the central carbon metabolic pathways along berry development under control and carbon limited conditions. The central carbon metabolic pathways are the same as in Fig. 1. Y-axis is in nmol min^-1^ per berry all for enzymes and is in FPKM for mRNA quantities. CL, carbon limited. Vertical bars indicate SE (*n=*3). The abbreviations represent the same enzymes and corresponding EC numbers as in Fig. 3. * indicates *P* < 0.05 and ** indicates *P* < 0.01.


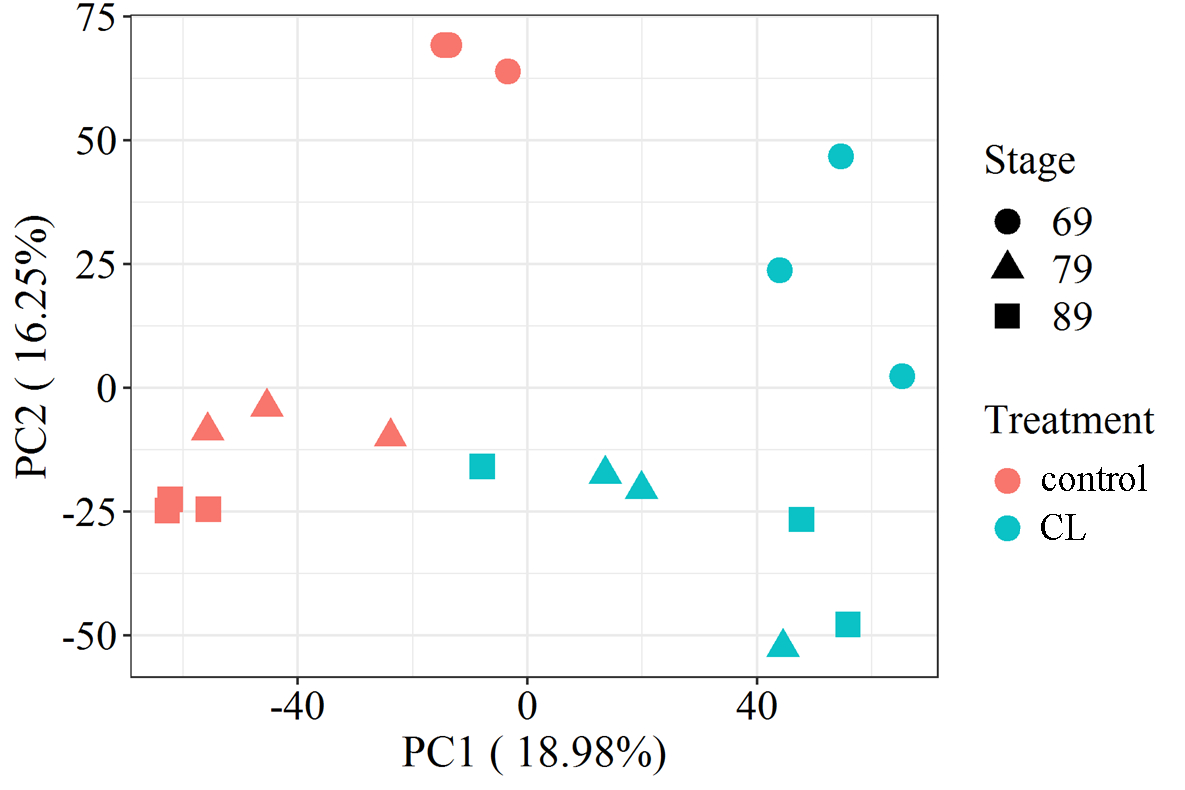


**Supplementary Figure S6**. Principal component analysis (PCA) of grape berries under control and carbon limitation (CL) during different development stage using RNAseq analysis (*n*=3).


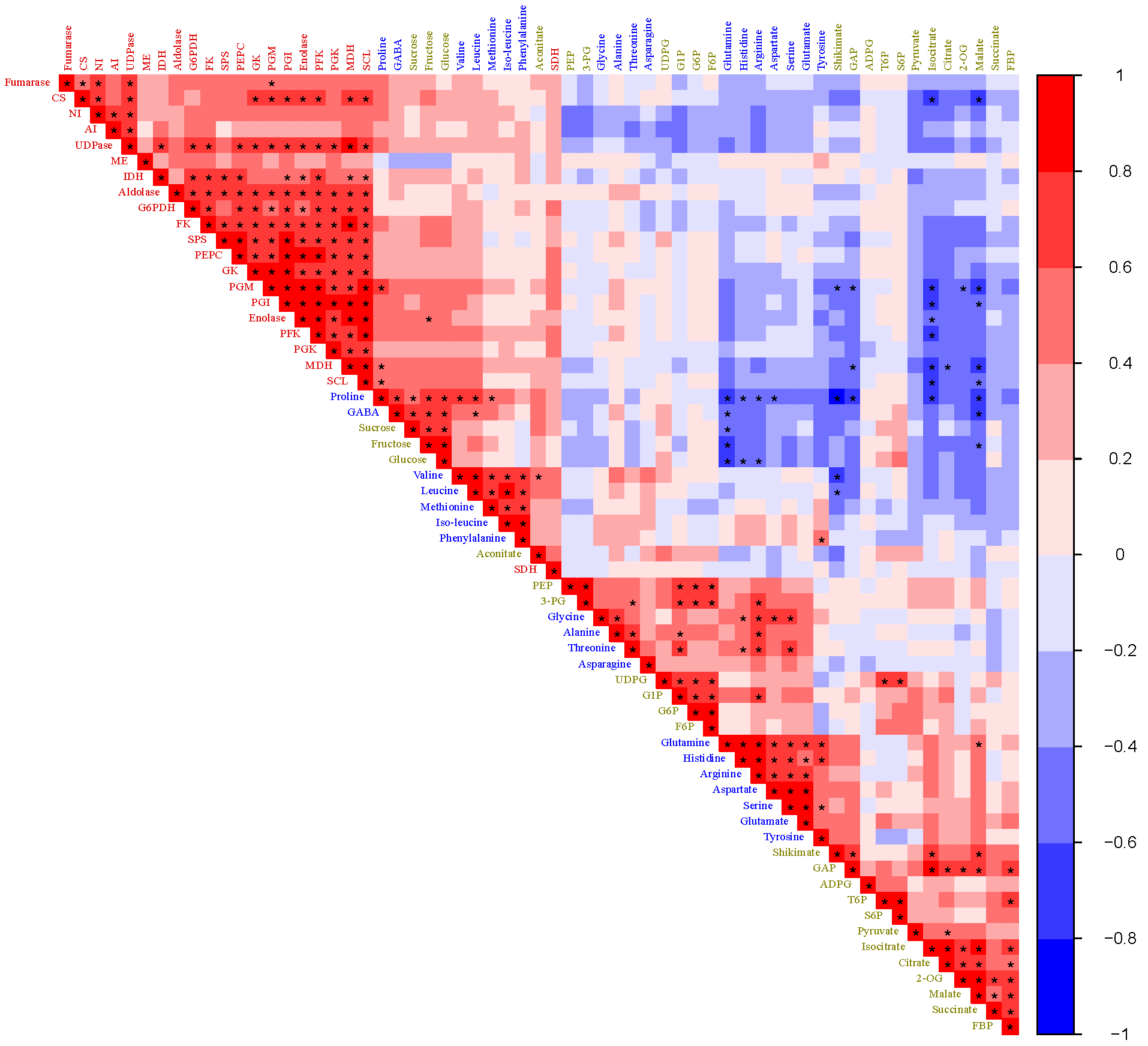


**Supplementary Figure S7**. Correlation matrix of metabolites and enzyme activities at nine developmental stages with pooling data from both carbon limitation (CL) and control berries (*n=*3).
